# Utility guideline and considerations for the novel Hugo™ RAS (robotic-assisted surgery) system in colorectal surgery: surgical outcomes and initial experience in a tertiary center

**DOI:** 10.1007/s00384-024-04715-7

**Published:** 2024-09-18

**Authors:** Antonio Arroyo, Ana Sánchez-Romero, Álvaro Soler-Silva, Saray Quinto, Francisco López-Rodríguez-Arias, María-José Alcaide, Mónica Serrano-Navidad, Elena Miranda, José-Luis Muñoz, Luis Sánchez-Guillén

**Affiliations:** 1 Colorectal Unit, Department of General Surgery, Elche University Hospital, Elche (Alicante), Spain; 2 Department of Pathology and Surgery, Miguel Hernández University, San Juan de Alicante (Alicante), Spain; 3 Department of Anesthesiology, Elche University Hospital, Elche (Alicante), Spain

**Keywords:** Robotic surgery, Colorectal surgery, Hugo™ RAS system, Ergonomics, Surgical innovations

## Abstract

**Purpose:**

A novel robotic platform—Hugo™ RAS (robotic-assisted surgery) system—has been introduced with several innovations that may prove advantageous for surgeons, such as an open console and four interchangeable modular arms. Our study aims to evaluate this platform’s safety, efficacy, and potential impact on the surgical treatment of colorectal pathology.

**Methods:**

Patients underwent robotic-assisted colorectal procedures with the Hugo™ RAS system at the General University Hospital of Elche from October 2023 to July 2024. Patient characteristics, intraoperative and postoperative variables, and robotic technical issues were recorded.

**Results:**

Forty consecutive patients were included (14 right, 13 left, and 8 rectum neoplasms; 4 left diverticulitis; and 1 ileocecal Crohn’s disease). The patients’ characteristics were as follows: median age, 69.5 years; 24 males and 16 females; 45% ASA III–IV; and Charlson Comorbidity Index > 5:42.5%.

We recorded four medical (2 anemia, 1 phlebitis, and 1 admission to the intensive care unit) and three surgical (1 hematoma of the incision, 1 intestinal occlusion, and 1 dehiscence of the anastomosis) postoperative complications. We had no conversions neither open nor laparoscopic surgery. The average hospital stay was 3 days, with no mortality or readmission.

**Conclusions:**

The Hugo™ RAS system is safe and feasible for colorectal procedures. The modularity of the arms provides the versatility of configurations adjusted depending on the patient’s body features and the surgeon’s preferences and greater adaptability to operating rooms. The open console is highly comfortable and ergonomic for the surgeon, allowing communication with the operating room environment.

**Trial registration:**

NCT06512480

**Supplementary Information:**

The online version contains supplementary material available at 10.1007/s00384-024-04715-7.

## Introduction

Robotic-assisted surgery has emerged as a promising minimally invasive technique for enhancing patient care in the management of colorectal cancer. It offers several advantages over laparoscopic procedures, particularly in technically challenging situations [1, 2]. Robotic surgery allows better accessibility and handling of surgical instruments for precise dissection, intracorporeal suturing, and high-vessel ligation, contributing to improved outcomes and patient well-being. However, the challenge remains to maintain the oncological standards of open and laparoscopic surgery with new robotic platforms.

The Da Vinci Xi system has emerged as the most prevalent robotic surgery platform on the European continent; however, the Hugo™ RAS (robotic-assisted surgery) system (Medtronic, Minneapolis, MN, USA) obtained CE marking in October 2021 for urological and gynecological procedures and in October 2022 for general surgery and has become a new and promising alternative. This novel platform has introduced several innovations that may prove advantageous for surgeons, including an open console and four interchangeable modular arms. The safety and feasibility of new robotic platforms play pivotal roles in shaping modern surgical practices. The first cases were published with this robotic platform for urological [3], gynecological [4, 5], bariatric [6], adrenal [7], and, recently, colorectal [8] procedures. Despite the consolidated results in urological surgery, there remains little clinical evidence in general surgery procedures, specifically colorectal surgery.

Our study aims to share the first results from colorectal surgery cases with the Hugo™ RAS system, which sheds light on the platform’s safety, efficacy, and potential impact on surgical practice.

## Materials and methods

### Patients

A case series involving robotic-assisted colorectal procedures with the Hugo™ RAS system at the General University Hospital of Elche, a referral center for colorectal surgery with extensive experience in laparoscopic surgery and no experience in robotics, was conducted from October 2023 to July 2024. No specific exclusion criteria were applied for patient selection. Two surgeons and one nurse had previously completed the technical training [9] on the platform delivered by Medtronic at ORSI Academy (Aalst, Belgium) and performed eight cholecystectomies with the same system to complete the training. Additionally, two assistant surgeons and five nurses completed the training and obtained certification. The first intervention was personally supervised by a robotic surgery expert, whereas later procedures were independently performed with onsite support from Medtronic technical staff. Informed consent was obtained from all participating patients.

### Data collection

The data were included anonymously in a computer database. For each patient, demographic, clinical, and perioperative data, prognostic comorbidity according Charles score [10], intraoperative robotics platform details, postoperative complications according to Clavien-Dindo classification [11], length of hospital stay, tumor stage (TNM), and follow-up data at 1 month were collected. Surgeries were recorded by Digital Surgery 1 (DS1) system of the Touch Surgery™ Ecosystem.

For statistical analysis, SPSS version 29.0 was used. For the descriptive analysis, appropriate estimators were used according to the type of variables means or medians, standard deviations or ranges for the quantitative variables, and percentages for the qualitative variables.

Ethical approval was obtained from the ethical committee of the General University Hospital of Elche (PI 60/2024)*.* The manuscript adheres to the STROBE guidelines for reporting observational studies.

### Preoperative protocol

Patients undergo an outpatient trimodal prehabilitation program with recommendations for aerobic and anaerobic physical exercise, nutritional supplementation (> 1.5 mg/kg/day protein; Fresubin® Protein Energy Drink, Fresenius-Kabi), and relaxation exercises for 30 days before surgery and the initial 30 days after hospital discharge.

### Patient position, trocar placement setup, and surgical technique

The Hugo™ RAS system consists of four independent arm carts. Two main settings are required to configure each arm. One is the tilt angle, which is the vertical angle of the arm with respect to the flat operative bed (0°) and can be adjusted by moving upward or downward from the arm’s distal section (Online Resource 1). The other is the docking angle, which is the clockwise horizontal angle between the head of the patient (0°) and the arm’s direction (Online Resource 2). Configurations were defined by our team along with the company’s recommended setup guides before the operations. Before docking the robotic arms, it is crucial to ensure that the lowest trocar is positioned at a height of at least 70% (or higher) of the numeric scale displayed on each cart.

**Fig. 1:**
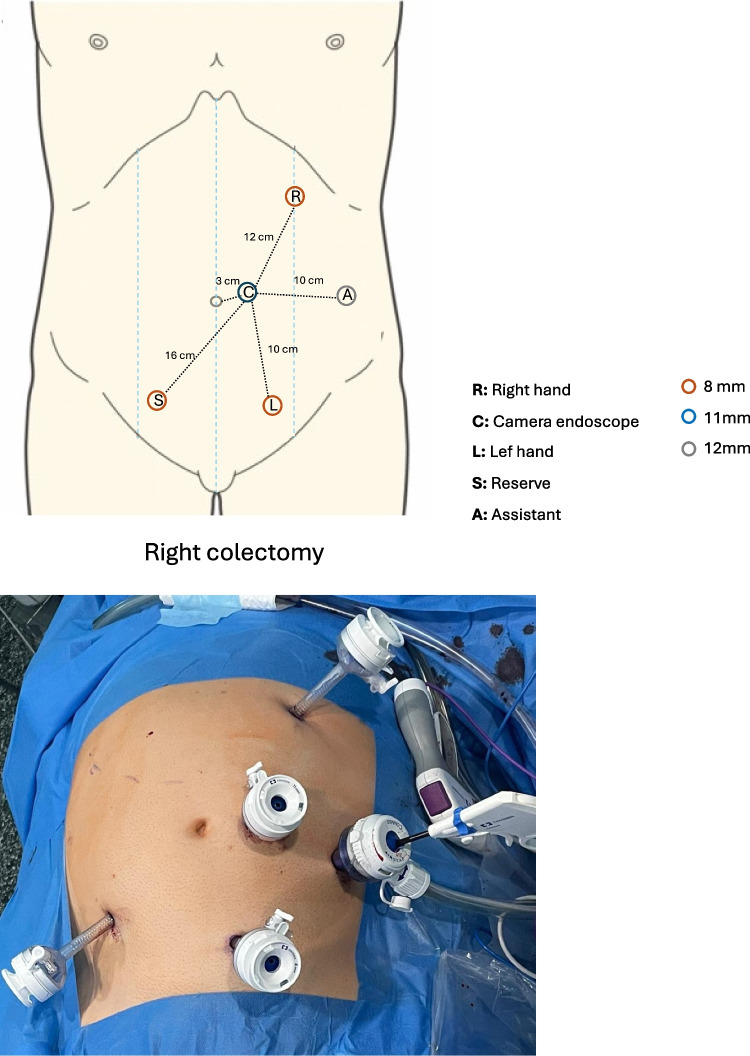
Trocar placement sites for robot-assisted right colectomy with the Hugo™ RAS system

**Fig. 2:**
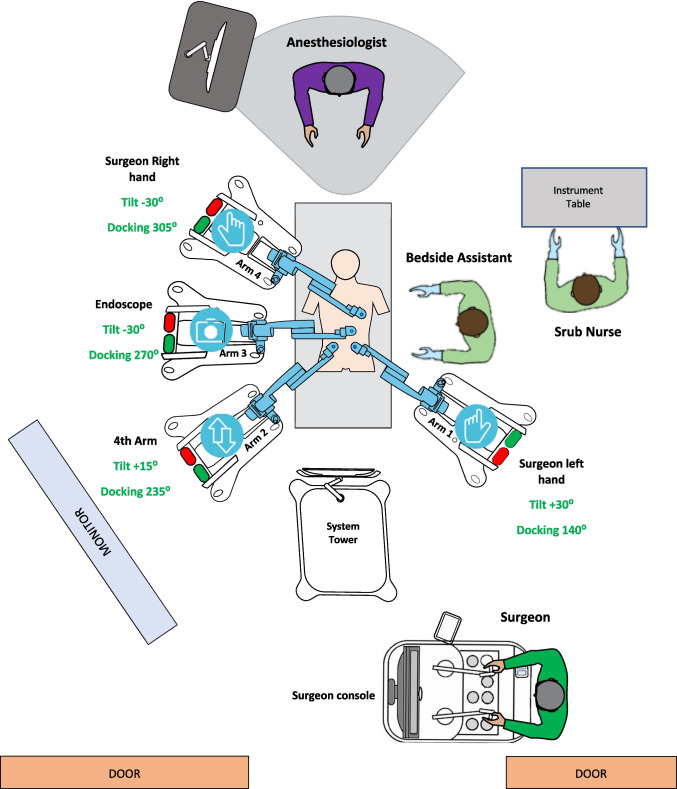
Operative setting for robot-assisted right colectomy with the Hugo™ RAS system

- Right colectomy:

The patient was placed in a supine position with the legs closed and arms tucked (or only the left arm opened). The pressure points and bony prominences were protected and secured with a gel pad. After the induction of the pneumoperitoneum (Veress needle) with the Airseal system® at Palmer’s point, an 11-mm optical port was placed in the left pararectal area 3 cm above the belly button. Trocar placement for right colectomy is shown in Fig. 1. A minimum distance of 8 cm between them was required to avoid collisions. The surgical table was placed in the Trendelenburg position with an angle > 20° and left side down with an angle of 10°–15°. The docking angles and tilt angles are shown in Fig. 2. The placement of the arm and docking maneuver is shown in Online Resource 3 and 4.

In total, a robotic right colectomy was performed via a medial-to-lateral approach. The ileocolic vessels (and, if necessary, middle colic vessels) were dissected with robotic scissors and sectioned at the level of the duodenum with Hem‐o‐Locks (Online Resource 5) or a 45 mm linear stapler (Signia™□) through the assistant trocar. The surgery was assisted with LigaSure through the assistant trocar.

The extracorporeal anastomosis was performed through a small 6 cm subcostal laparotomy with a dual-ring wound protector (Alexis® retractor) and an end-to-lateral ileocolic anastomosis via a circular stapler (EEA Circular Stapler with Tri-Staple™□ technology, 28 mm, Medtronic). A 60-mm lineal stapler (Signia™□, Medtronic, beige staple vascular/medium) was used to transect the colon. For intracorporeal anastomosis, both the ileum and the colon were transected with a stapler for a subsequent side-to-side anastomosis with a Signia™□ (60 mm beige Tri-Staple™□ vascular/medium) (Online Resource 6), and the enterotomy was closed with a continuous 3/0 barbed suture of resorbable material (Online Resource 7). For intracorporeal anastomosis, the sample was removed through a Pfannenstiel incision protected with an Alexis® protector.

**Fig. 3:**
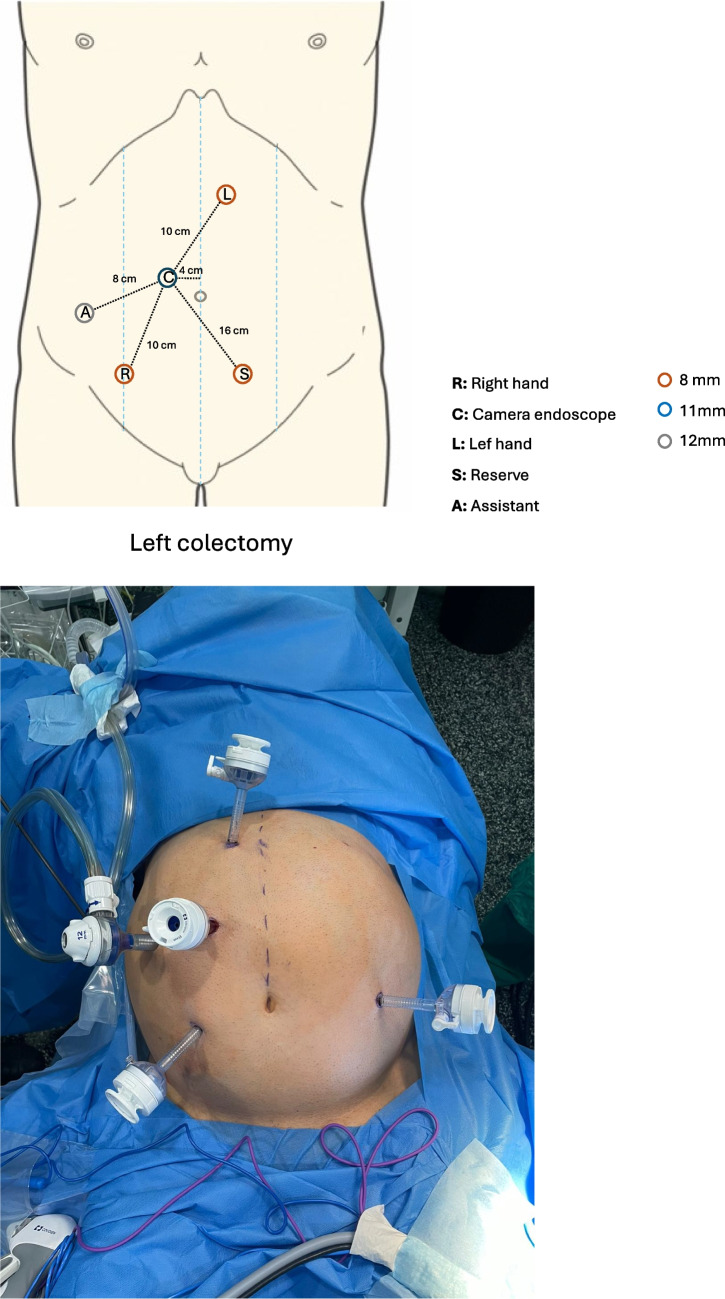
Trocar placement sites for robot-assisted left colectomy with the Hugo™ RAS system

**Fig. 4:**
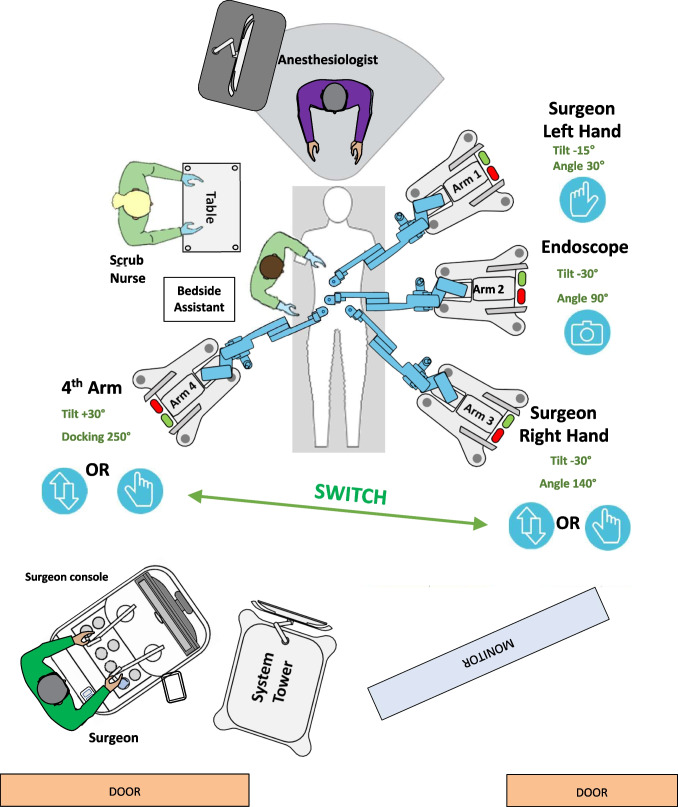
Operative setting for robot-assisted left colectomy with the Hugo™ RAS system

- Left colectomy/sigmoidectomy/anterior rectal resection:

Patients in a modified lithotomy position with their arms tucked (or only the right arm opened) and legs in adjustable stirrups. After induction of the pneumoperitoneum, an 11-mm optical port was positioned in the right pararectal area 4 cm from the belly button. Trocar placement for left colectomy is shown in Fig. 3. The surgical table was tilted in a Trendelenburg position > 20°, and the right tilt angle was 10–15°. The docking angle and tilt angle positions are shown in Fig. 4.

In total, a robotic left colectomy was performed via the medial-to-lateral approach. The surgery was assisted with LigaSure through the assistant trocar (Online Resource 8). Using robotic scissors, the inferior mesenteric vessels were dissected and sectioned with Hem‐o‐Locks or a 45 mm lineal Tri-Staple™□ (Signia™□) (Online Resource 9) through the assistant trocar.

The sample was removed through a Pfannenstiel incision protected with an Alexis® protector. Anastomosis was performed with a circular stapler (EEA Circular Stapler with Tri-Staple™□ technology, 31 mm, Medtronic) (Online Resource 10) and air-checked for leaks.

We didn’t perform a systematic complete splenic flexure takedown. To descend the splenic flexure, we can optionally change the patient’s position (anti-Trendelenburg) by performing previous undocking and modifying the tilt of the arm, with minimal modification of the degrees of the arms and maintaining the position of the trocars. In this case, the instruments on arm 3 (grasper) and arm 4 (scissor) are exchanged (Online Resource 11).

**Fig. 5:**
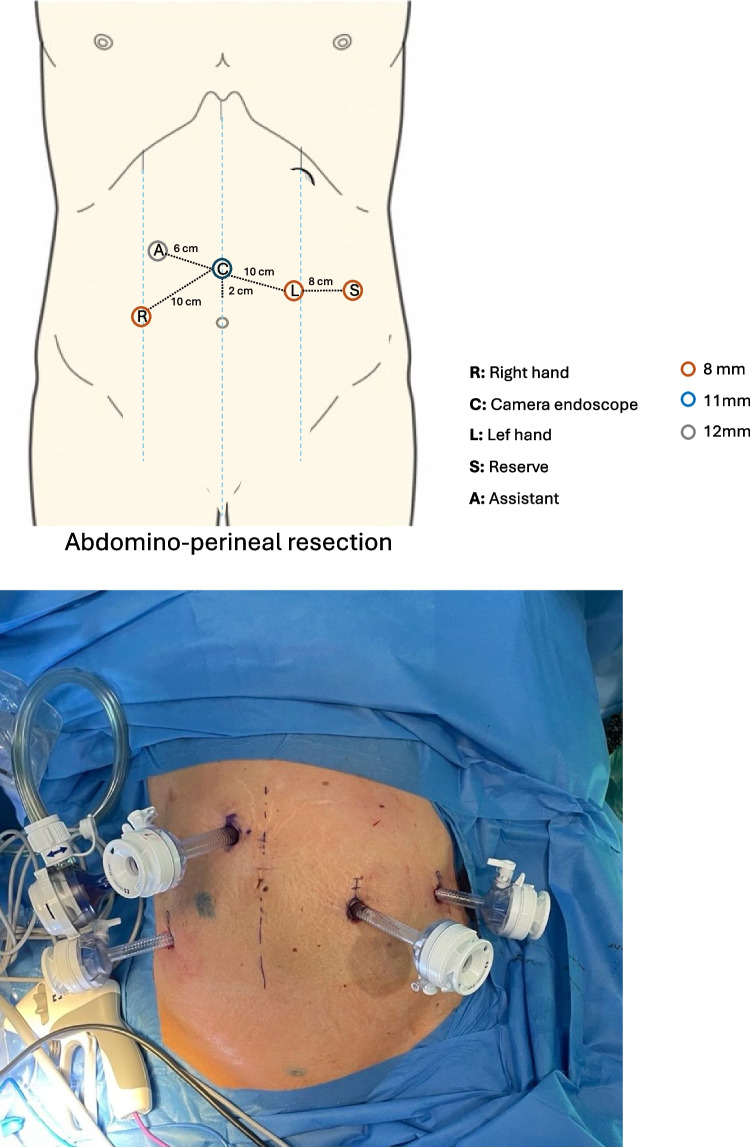
Trocar placement sites for robot-assisted rectal resection with the Hugo™ RAS system

**Fig. 6:**
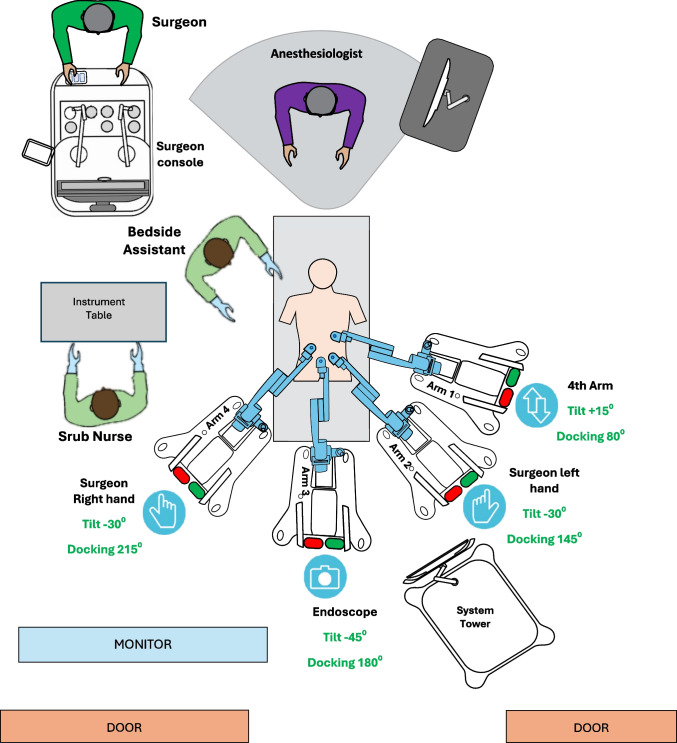
Operative setting for robot-assisted rectum resection with the Hugo™ RAS system

- Low rectum resection:

Patients in a modified lithotomy position with their arms tucked (or only the right arm opened) and legs in adjustable stirrups. After induction of the pneumoperitoneum, an 11-mm optical port was placed in the right belly button area. Trocar placement for rectum resection is shown in Fig. 5. The surgical table was tilted in a Trendelenburg position > 15°, and the right tilt was 5–15°. The docking angle and tilt angle positions are shown in Fig. 6.

The steps of the procedure were like those of sigmoid resection. Perineal time in cases of abdominoperineal resection (APR) was conducted conventionally, along with the sample extraction.

### Postoperative protocol

An ERAS protocol [12] identical to that used in laparoscopic surgery was used.

## Results

### Patient outcomes

Forty consecutive patients were included. The patient characteristics, as well as perioperative data, are summarized in Table 1. The median age was 69.5 years (22–89 years), with 24 male and 16 female patients. A total of 45% of 17 patients (42.5%) had ASA statuses III–IV and “ > 5” (high comorbidity) according to the Charlson Comorbidity Index (CCI), which calculates 1-year comorbidity and mortality and predicts 10-year survival in patients with multiple comorbidities. A total of 14 right colon tumors, 13 left colon/sigmoid neoplasms, 8 rectal neoplasms, 4 cases of left diverticulitis, and 1 case of ileocecal Crohn’s disease were treated.

**Table 1:**
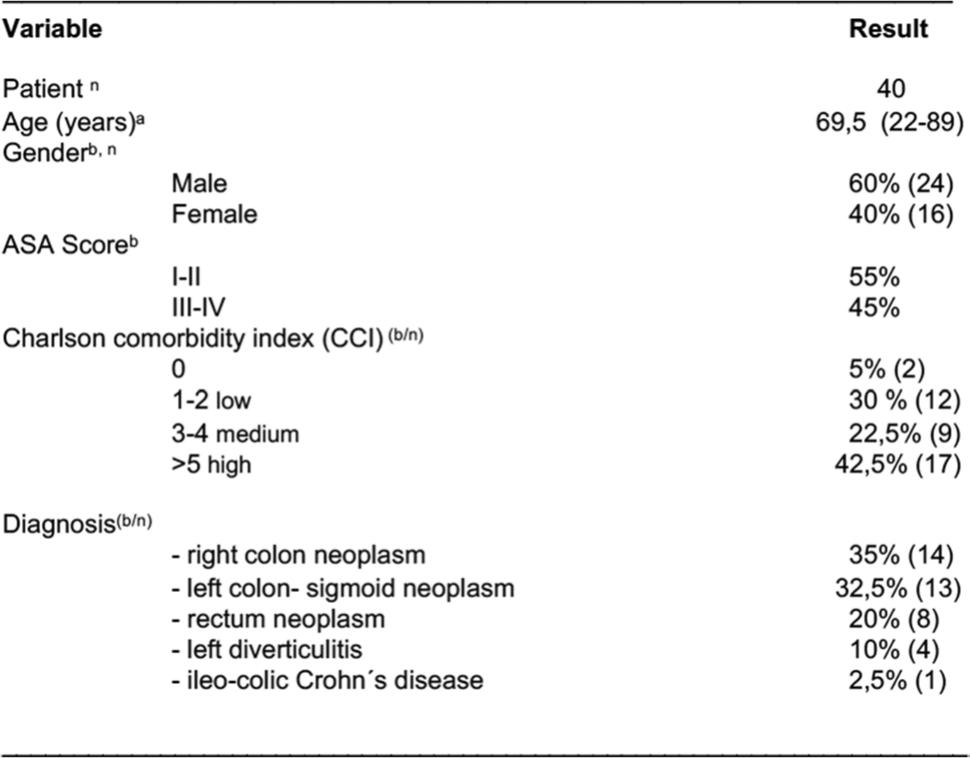
Characteristics of patients who underwent colorectal resections with Hugo™ RAS

^a^values are presented as median (interquartile range)

^b^values are presented as %

*n* number of patients

*ASA* American Society of Anesthesiologists, *CCI* Charlson comorbidity index

Predicts 10-year survival in patients with multiple comorbidities

The types of surgical procedures and Hugo robot times in the different phases of surgery are described in Table 2. The first 12 right anastomoses were performed extracorporeally (usual clinical practice in our unit), with three intracorporeal anastomoses. A right colectomy could be performed without modifying the initial patient’s configuration and position. In only two patients—the left and rectum colon—due to the descent of the splenic flexure, we had to change the patient’s position (anti-Trendelenburg) by undocking and modifying the tilt of the arm. We had no conversions neither open nor laparoscopic surgery and placed five drains (all in the rectum).

**Table 2:**
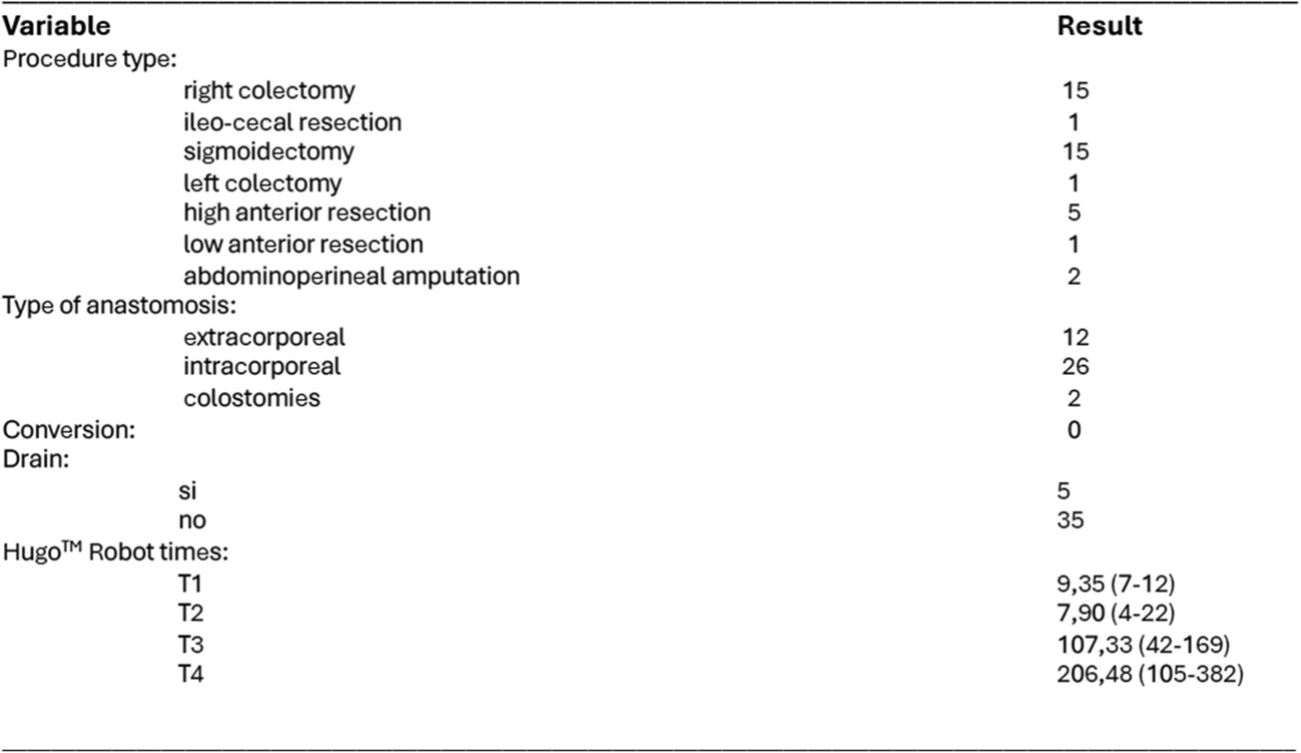
Outcomes of intraoperative characteristics of patients treated with colorectal resection with Hugo™ RAS

*n* number of patients

T1: drapping time: mean minutes and (range)

T2: docking time: mean minutes and (range)

T3: consola time: mean minutes and (range)

T4: total surgery time (skin to skin time): media minutes and (range)

With respect to postoperative morbidity (Table 3), we recorded four medical complications: 2 cases of anemia that required transfusion (Clavien‒Dindo 2), 1 case of phlebitis (Clavien‒Dindo 1), and 1 case of admission to the intensive care unit due to the need for vasoactive drugs because of patient morbidity. We observed three surgical complications: 1 hematoma of the Pfannenstiel incision (Clavien–Dindo 2), which did not require surgical treatment, and 2 intestinal occlusions in two cases of sigmoidectomy (Clavien–Dindo 3), which required surgical treatment. Occlusion was due to adhesion of the small intestine to the dissection area, and only release was necessary; the other type of occlusion was caused by dehiscence of the anastomosis, which required resection of the anastomosis and colostomy. We obtained an average stay of 3 days with no mortality or readmissions. No mortality was recorded. In the pathological analysis, no tumor perforations were recorded, the median number of lymph nodes extracted was 17, and all the mesorectal excisions were completed.

**Table 3:**
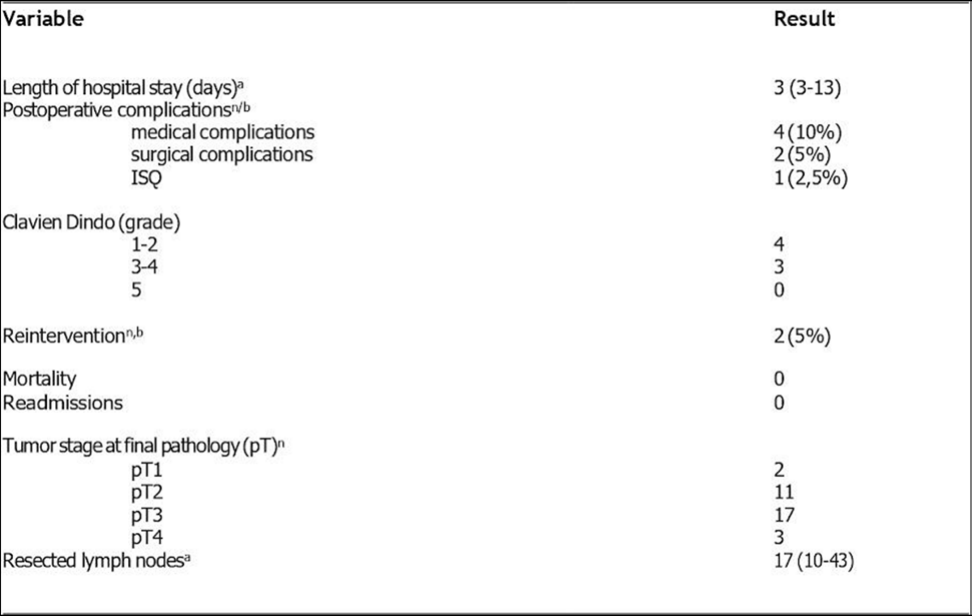
Outcomes of postoperative characteristics of patients treated with colorectal resection with Hugo™ RAS

^a^values are presented as median (interquartile range IQR)

^b^values are presented as %

*n* number of patients

### Robotic technical issues during implementation

All procedures were performed by one console surgeon with one bedside assistant and two trained nurses. There were no injuries to the patients related to the device. Minimal collisions of the arms that did not cause interruptions in the surgery took place during the first cases. These were corrected with minimal modifications of the tilt/degrees of the affected arms during the surgery by the bed assistants and minimized with the learning of the movements of the instruments by the console surgeon. Among the instruments used for the procedures, only one scissor had to be replaced. In two cases, during platform power-up, one arm was not recognized. Once was solved by restarting the system, and in the other, the surgery was performed with only 3 robotic arms without problems. A failure in a component of the arm was detected, and the Medtronic Field Service Engineer repaired it in 48 h. There were no problems with DS1 or the transfer of videos.

## Discussion

Implementing the robotic platform Hugo™ RAS system for colorectal surgery seems feasible and safe, with adequate oncological and postoperative results for all patients in our group, who had extensive laparoscopic experience but were naive to robotic surgery.

To our knowledge, this is the longest published series of colorectal resections with the Hugo™ RAS system. Our coloproctology unit is accredited by the Spanish Association of Coloproctology (AECP) as an advanced unit in Spain that achieves quality standards in colorectal surgery [13]. Among them, the outcome standards for patients with colorectal cancer (surgical site infection < 12.5%; anastomotic leakage < 5%; mortality < 7%; readmission < 5%; reintervention < 6%; stay < 6 days) continue to be met in our series of procedures with this robotic platform. Therefore, our study confirms the good initial results of very few cases, [8, 14–17] who reported that the Hugo™ RAS system is safe and feasible for colorectal procedures.

To date, only two long series of cases in which major colorectal surgery were performed safely and effectively with Hugo RAS have been published. The first study by Romero et al. [9], with ten cases—five right colectomies, three sigmoid resections, one high rectal resection and one ventral mesh rectopexy—reported no cases of anastomotic dehiscence or conversion to laparoscopy, with a hospital stay of 3 days. The second study by Belyaev et al. [18] included 31 cases—11 sigmoid, nine rectal, eight right, one left cancers, and two Hartmann’s reversal—reported two conversions due to adhesions: one anastomosis leak, one paralytic ileus, two presacral hematomas, and one medical complication with a hospital stay of 8 days.

Studies on urogynecological procedures performed with this platform have also demonstrated its safety and effectiveness [3–7]. In fact, in robotic prostatectomy and nephrectomy, where there is greater experience with Hugo™ RAS than with colorectal surgery, comparisons with the Da Vinci system have yielded comparable results [19, 20].

The Hugo RAS robotic platform consists of four interchangeable modular arms and an open console, which are the main differentiating characteristics from the Da Vinci platform. The first great advantage of this robotic platform is the modularity of the different arms of the system, which allows configurations or setups that can be slightly adjusted depending on the patient's body features and the surgeon’s individual preferences [9, 14], just as it performs in laparoscopic surgery. This greater versatility compared with unimodular robotic platforms is especially relevant in surgeries of different quadrant areas since it allows a greater range of mobility and access to multiple quadrants without the need to insert new trocars or change the configuration. In our series, in only two patients due to the descent of the splenic flexure, we had to change the patient’s position (anti-Trendelenburg) by undocking and modifying the tilt and angles of the arms in a comfortable and fast way without moving the position of the arm carts. A right colectomy can be performed without modifying the initial patient’s configuration and position. It is really exciting and we were surprised from the first right colectomy by the ease of being able to approach the transverse colon and lymphadenectomy without having to change either the patient’s position (from Trendelenburg to anti-Trendelenburg) or the configuration of the arms. Without a doubt, a correct setup based on the patient’s characteristics and the different anatomical areas in which we will perform the surgery is essential. In the case of extended right colectomies, we recommend lateralizing the trocars to the left, somewhat further from the midline for access to the transverse colon. The modularity of the robot allows us to play with these aspects.

In contrast, owing to the need to bring four carts closer to the surgery table, even the initial docking times described with this platform are relatively quick—7 min in our experience, 14 min [9], 12 min [18], 5 min [14], and 8 min [8]—and easy to perform, which, together with the mobility of each robotic arm throughout the operating room, allows a change back to robotic surgery after laparoscopic surgery and vice versa without problems, such as emergent bleeding or some steps of difficult cases.

Even its multimodular feature grants it certain adaptability in operating rooms whose configuration is not recommended for installing unimodular robotic platforms owing to size and weight restrictions. In our hospital, a previous weight-bearing study indicated that the Da Vinci robotic platform could not be installed without floor reinforcement and that some hospitals could have problems due to insufficient height of the operating rooms. However, the Hugo™ RAS platform could be installed given the weight/m2 distributions of the different modular components of the robot. Furthermore, modularity allows for quick, safe, and easy transport of components within the operating room and even mobility between different operating rooms.

However, the separated placement of the four arms carts around the operating table takes up a lot of space and cause more spatial constraints in operating rooms than the Da Vinci platform. This fact limits the correct positioning of the assisting surgeon, which is especially relevant at this time in the absence of stapling devices and energy instruments integrated into the Hugo platform.

For the open console display system, the second major difference to the Da Vinci robotic platform is that vision is achieved through a 3D glasses system with eye tracking, providing a security blocking system. The vision is magnified by 10 × , which offers, in our opinion, the fact that we were users of 3D laparoscopic towers with the same Stroz system, a surprisingly better and spectacular vision compared with this system, which produces a truly immersive vision of the surgical field. Compared with closed immersive platforms, the open console significantly improved communication between the surgeon and the operating room team. This direct flow of communication potentially impacts complex intraoperative situations. The open console offers quick visualization of the patient’s position with the robotic arms during the procedure, which is vitally important during the learning curve and in cases with multiquadrant access. On the other hand, one of the most important aspects of a university hospital such as ours is that the open console facilitates the teaching system since it does not require an additional console to visualize the surgical field and movements, thus improving training and teaching during the same procedure. In addition, the open system is very comfortable and ergonomic for the surgeon since it enables a sitting position in a chair with a backrest and the possibility of more natural body movement. Improved ergonomics and comfort could mitigate occupational injuries and extend the longevity of surgeons’ careers [21, 22].

The Hugo™ RAS system incorporates the Touch Surgery™ DS1 Video solution, the first smart surgical video recorder on the market [18]. Equipped with real-time artificial intelligence (AI), the device can capture, anonymize, and upload sensitive surgical footage automatically to the cloud. This enables surgeons to monitor and review their performance and share footage securely with others in their field, supporting surgeon learning and improving patient outcomes. In addition, DS1 can incorporate an accessory module with an additional external camera for real-time streaming of OR, surgeon, and surgical videos to external users through their phones or tablets and personal computers with two-way interaction. This tool represents a milestone for the remote proctorization of users, teaching and training tasks, and the live broadcast of surgeries at events.

Although the Hugo™ RAS system is fully operational, it is under continuous development and integration into new surgical procedures. In terms of instrumentation, advanced bipolar energy systems such as LigaSure™□ or robotic Tri-Staple™□ and the integration of indocyanine green will soon be available for this platform. Hybrid surgery is necessary through an accessory port used by the assistant surgeon.

In conclusion, Hugo™ RAS is a valuable addition to robotic surgery, addressing Da Vinci robotic platform limitations with its open console, enhanced optics, and tetrameric structure. The new modular platforms are anticipated to provide increased versatility, eliminating the need for adapted operating rooms and allowing flexible placement of robotic arms around the patient. Incorporating open consoles and polarized glasses enhances the sense of freedom and ergonomics. However, it remains to be proven whether the advantages historically associated with the da Vinci system over conventional laparoscopic approaches persist when these emerging platforms are utilized. Further research is needed to validate and compare the performance of this new system in various surgical scenarios.

## Supplementary Information

Below is the link to the electronic supplementary material.

Supplementary file1 (MP4 2074 KB)

Supplementary file2 (MP4 5261 KB)

Supplementary file3 (MP4 5346 KB)

Supplementary file4 (MP4 7216 KB)

Supplementary file5 (MP4 25901 KB)

Supplementary file6 (MP4 40032 KB)

Supplementary file7 (MP4 495801 KB)

Supplementary file8 (MP4 30278 KB)

Supplementary file9 (MP4 85259 KB)

Supplementary file10 (MP4 152968 KB)

Supplementary file11 (MP4 2073 KB)

## Data Availability

The data that support the findings of this study are available from the corresponding author upon reasonable request.
